# Mechanotransduction as an Adaptation to Gravity

**DOI:** 10.3389/fped.2016.00140

**Published:** 2016-12-26

**Authors:** Tanbir Najrana, Juan Sanchez-Esteban

**Affiliations:** ^1^Department of Pediatrics, Alpert Medical School of Brown University, Women & Infants Hospital of Rhode Island, Providence, RI, USA

**Keywords:** gravity, earth (planet), mechanotranduction, cytoskeleton, tensegrity, microgravity, genomics, epigenetic

## Abstract

Gravity has played a critical role in the development of terrestrial life. A key event in evolution has been the development of mechanisms to sense and transduce gravitational force into biological signals. The objective of this manuscript is to review how living organisms on Earth use mechanotransduction as an adaptation to gravity. Certain cells have evolved specialized structures, such as otoliths in hair cells of the inner ear and statoliths in plants, to respond directly to the force of gravity. By conducting studies in the reduced gravity of spaceflight (microgravity) or simulating microgravity in the laboratory, we have gained insights into how gravity might have changed life on Earth. We review how microgravity affects prokaryotic and eukaryotic cells at the cellular and molecular levels. Genomic studies in yeast have identified changes in genes involved in budding, cell polarity, and cell separation regulated by Ras, PI3K, and TOR signaling pathways. Moreover, transcriptomic analysis of late pregnant rats have revealed that microgravity affects genes that regulate circadian clocks, activate mechanotransduction pathways, and induce changes in immune response, metabolism, and cells proliferation. Importantly, these studies identified genes that modify chromatin structure and methylation, suggesting that long-term adaptation to gravity may be mediated by epigenetic modifications. Given that gravity represents a modification in mechanical stresses encounter by the cells, the tensegrity model of cytoskeletal architecture provides an excellent paradigm to explain how changes in the balance of forces, which are transmitted across transmembrane receptors and cytoskeleton, can influence intracellular signaling pathways and gene expression.

## Introduction

Life evolved from the sea. Organisms borne in the primitive sea about 30 million years ago had evolved in water without a large influence of gravity on earth. Underwater, gravity is almost compensated by buoyancy, “the upward force exerted by a fluid that opposes the weight of an immersed object.” In contrast, other forces such as drag and lift became significant in an aquatic environment. These flow forces vary in magnitude and direction, and under specific conditions, can even reach higher values than gravity. In addition, viscous forces and the lack of surface tension under immersed conditions are also important in the aquatic environment (1). Some aquatic species may still sense gravity. However, the pressure around them and the internal organs are able to counterbalance the force of gravity (2). For example, the swim bladder, the evolutionarily homologous to the lungs, is an internal gas-filled organ of many bony fish. It has flexible walls that contract or expand according to the ambient pressure to obtain neutral buoyancy and ascend and descend to a large range of depths. However, some aquatic species appear to use gravity as a directional cue. Throughout evolution, aquatic species have adapted to their environment. In addition to inner ear hair cells, aquatic vertebrates have hair cells on the surface of their body in the lateral line system. These mechanoreceptors provide a highly sensitive and versatile hydrodynamic sense that is used in a wide range of behavior (3). In order to understand how these receptors mechanically filter signals, a theoretical model of the superficial neuromast in the fish lateral line system has been developed (4). Other species such as crustaceans contain a great variety of sensilla along their antennules that enable them to sense both hydrodynamic and chemical stimuli in aquatic environments (3).

In spite of the more or less constant gravitational force on Earth, mechanical load on organisms on land is approximately 1,000 times larger than in water. About 4 million years ago, the first terrestrial organisms, plants appeared on the land from the sea. The terrestrial plants have adapted to and evolved on the land environment, so that they can extend their roots downward in soil and their shoots upward against 1 × *g*. Land species began to develop adaptive mechanisms to orient themselves to the gravity vector.

An attractive hypothesis that gravity might have played a key role in evolution comes from studies in snakes (5). On Earth, snakes live in different environments. For example, tree snakes crawl up trees and have to cope with gravity when compared to land snakes that spend most of their life in a horizontal position or even the neutrally buoyant sea snakes. Lillywhite found out that the heart of the tree snake was closer to the brain when compared to the other snakes. These studies indicate that even without altering the magnitude of gravity, changes in orientation with respect to the direction of a gravitational force may have played an important role in the evolution of the species on Earth. In this specific example, gravity may have determined the location of the heart and perhaps other internal organs in snakes.

What about species that “returned to the water” as descendants from non-aquatic ancestors, like cetaceans? The origin of whales is one of the best examples of macro-evolutionary change in vertebrates. As they became obligated marine, they had to adapt to the new environment. Studies from fossil suggest that this adaptation took less than 15 million years and different organs followed distinct evolutionary patterns. Although the anatomy and function of the sensory organ differ among them, all take advantage of the non-compressive and dense nature of the water and fossil anatomic analysis of early cetaceans showing parts of the snout of early cetaceans show extreme pitting and grooving. These findings suggest that the tip of the snout was important as mechanoreceptor. In addition, during aquatic adaptation, some organs involved in olfaction, vomeronasal sense, and balance underwent an involution process (6). Another example of evolution in cetaceans is the hearing sense. The ear of whales, initially intended for hearing on the surface, became adapted for hearing underwater and was substituted by the mandible and the mandibular fat pad. “Pakicetids, the earliest archaeocetes, had a land mammal ear for hearing in air, and used bone conduction underwater. Remingtonocetids and protocetids were the first to display a genuine underwater ear where sound reached the inner ear through the mandibular fat pad. Basilosaurids and dorudontids showed further aquatic adaptations of the ossicular chain and the acoustic isolation of the ear complex from the skull” (7, 8).

Life on Earth has developed in the presence of gravity. Therefore, it is important to understand the effect of this force on the evolution of terrestrial life. “Although it is clear that physical forces, such as those due to gravity, are fundamental regulators of tissue development, little is known about how living cells sense these signals and convert them into a biochemical response. This transduction process is known as mechanotransduction” (9). Certain cells have evolved specialized structures, such as statoliths in plants and otoliths in hair cells of the inner ear, to respond directly to the force of gravity.

Gravity alterations experienced by astronauts have important effects on the human body. For example, when standing upright on Earth, fluids are accumulated in the lower extremities. In contrast, in microgravity, fluids are displaced to the thorax and head. As a result, there is an increase in cardiac filling and diuresis, and the overall effect is a reduced blood volume. In addition, microgravity reduces the loading of skeletal muscles leading to decreased muscle mass, muscle strength, and endurance. Moreover, low gravity increases loss of calcium from the bone and has an inhibitory effect on bone formation and subsequent vulnerability to fracture. On entering microgravity, removal of gravitational information results in altered body and spatial orientation and visuo-motor coordination (10).

Microgravity has also a significant impact on the physical forces encountered by cells. By performing studies in a reduced gravity environment, we may get better understanding on how gravity affected life on Earth. However, performing experiments in the space are difficult for obvious reasons. Alternatively, several ground-based models have been designed, including random positioning machine, diamagnetic levitation, and hind limb unloading. In addition, some cell culture systems, such as the clinostat and rotary cell culture system, also simulate microgravity (11).

*In vitro* studies have demonstrated that cell proliferation, growth, differentiation, signaling, shape, and gene expression are all altered by microgravity (12–14). However, the mechanisms responsible for this adaptation to changes in gravity remain controversial. This information is important not only for the effects of spaceflight at the tissue/organism level but also to study the response of cells to the decrease of force under microgravity (15).

In this manuscript, we will review first how individual cells (both prokaryotic and eukaryotic) are able to sense gravity; then, we will discuss how more complex eukaryotic tissues (plants, animals, developmental windows for programing) and organisms respond. Finally, the molecular mechanisms that are implicated in cytoskeletal signal transduction and the concept of tensegrity will be discussed.

### Bacteria

Previous investigations have shown that spaceflight increase the risk of infections in both humans and animals (16, 17). Experiments with various microbes have found that microgravity has a significant impact on several factors, such as growth, morphology, metabolism, genetic transfer, and viral reactivation (18). For example, it has been shown that bacterial growth increases by 88% in microgravity when compared to ground controls (19). Interestingly, they also observed an increased resistance of *S. aureus* and *Escherichia coli* to antibiotics (20). Simulated microgravity using the RWV has also observed an enhanced virulence of a Gram-negative bacterial pathogen, *Salmonella* Typhimurium (21). Typhimurium showed increased resistance to acid, to osmotic and thermal stresses, and an increased capacity to survive inside the macrophages, when compared to cells grown under normal gravity conditions (21–23). In conclusion, the ability of bacteria to proliferate and grow under low gravity conditions is enhanced. Therefore, there is an increased susceptibility to infections in microgravity (22).

In addition, microgravity seems to significantly affect gene expression in prokaryotes (24). For example, DNA microarrays were used to investigate changes in gene expression of *Salmonella* Typhimurium exposed to simulated microgravity. Compared to normal gravity conditions, simulated microgravity differentially regulated the expression of 163 genes encoding transcriptional factors, virulence factors, lipopolysaccharide biosynthetic enzymes, ribosomal proteins, iron utilization enzymes, etc. (23). Thus, microgravity is an important environmental factor able to reprogram gene expression (24).

### How Do Microbes Respond to Microgravity?

The response of a microbial cell to changes in extracellular solute concentration is critical for cellular function and survival. Microbes sense changes in osmotic pressure gradients *via* tension in the cell membrane (25, 26). Several studies have demonstrated that cells recognize changes in gravity at the cell surface (27, 28). Indeed, microgravity causes changes in the cell surface of microbial cells (23, 26–29). It has been shown that changes in fluid shear force can affect bacterial adhesion (30). Therefore, it appears that decreased distortion in the cell surface, caused by lower level of fluid shear forces, is an important mechanism for cellular mechanotransduction under microgravity conditions. Studies performed in *E. coli* FimH provide a model for mechanotransduction in microbes (27, 31). In this model, a sensor protein embedded in the prokaryotic cell membrane has two domains connected by a flexible linker region. One domain is embedded in the membrane and can serve to initiate signaling inside the cell. The other domain is extracellular and can initiate conformational changes in the linker in response to the environment.

### Yeast

Previous studies have examined the effect of microgravity on cell function, morphology, and genomic expression of the yeast *Saccharomyces cerevisiae*. With the exception of a shortened lag phase, yeast cells grown under microgravity conditions did not vary from the controls. However, they differed in the formation of polarity, as shown by abnormal budding compared to the bipolar pattern present in the controls. The aberrant budding was accompanied by an increased tendency of cells to clump (32).

Additional investigations using DNA microarray (33) found that 1,372 genes (36%) were significantly altered by exposure to low-shear modeled microgravity, from which 26% of them were environmental stress responses genes. Notably, one of the genes most dramatically affected was HSP30 which is known to be responsive to high hydrostatic pressure (34, 35). In addition, they also found significant changes in the expression of genes associated with the establishment of polarity, bipolar budding, and cell separation. Thus, low-shear environments may significantly alter yeast gene expression and phenotype, as well as evolutionary conserved cellular functions such as polarization.

Cell polarity refers to “spatial differences in the shape, structure, and function of cells.” Budding is a “form of asexual reproduction in which a new organism develops from an outgrowth or bud due to cell division at one particular site.” Normal yeast cells assemble buds in response to a well-orchestrated program that promotes polarized growth. Investigations performed in yeast exposed to simulated microgravity show remarkable differences in the establishment of cell polarity, bipolar budding pattern, and cell separation (32, 33). Specifically, they identified four genes involved in bud pattern selection (*BUD5, RAX1, RAX2*, and *BUD25*) and three genes related to cell separation (*DSE1, DSE2*, and *EGT2*). *BUD5* regulates the activity of the small GTPase Cdc42 and plays a key role in the initiation and establishment of cell polarity during budding (36). In addition to a random budding phenotype, cells grown in microgravity were also found in clumps containing five or more cells (32). *DSE1, DSE2*, and *EGT2* are part of a cell division program (37) and rely on the accuracy of the cell polarization process (38, 39). Thus, “the low microgravity-induced defect in cell polarity could be the underlying mechanism for both the random bud scarring and the formation of the large cellular aggregates observed” (32).

Yeast cells must maintain cell wall integrity when exposed to changes in the extracellular environment such as osmotic stress and oxidative stress (40). Moreover, during polarized cell growth, yeast cells have to preserve cell shape to avoid rupture during bud formation and subsequent cell division. The MAPK signaling pathway is primarily responsible to regulate changes to the cell wall under these conditions. Microarray analysis identified two protein phosphatases SDP1 and PTP2 upregulated by microgravity. Both proteins inhibit the MAPK signaling pathway. Therefore, downregulation of the MAP kinase by SD1 and PTP2 can compromise cell shape and polarity. “The changes in phenotype, together with the microarray results, implicate the cell wall integrity MAP kinase pathway as a possible mechanism for sensing the low shear microgravity environment” (33).

### Signaling Pathways Regulating Chemotaxis

Chemotaxis is defined as the movement of a motile cell or organism, or part of one, in a direction corresponding to a gradient of increasing or decreasing concentration of a particular substance. Directed cell migration and cell polarity are basic migratory systems that have been conserved throughout evolution (41–43). Cells have evolutionally developed a mechanism to respond to an extracellular gradient of chemoattractant *via* the PI3K signaling pathway. The PI3K family proteins are lipid kinases that phosphorylate phosphatidylinositol (PI) or phosphatidylinositol phosphate. PI3K is primarily responsible for the production of PI(3,4,5)P_3_ at the leading edge of the plasma membrane in response to extracellular stimulation (44).

Several studies in *Dictyostelium* have demonstrated that Ras regulates both directional movement and cell polarity (45, 46), but independently from each other, as shown in one of the RasGEF mutants Aimless, where *aimless* null cells show a reduced directionality without affecting cell polarity or chemotaxis speed (47, 48). Ras is quickly activated after stimulation in *Dictyostelium* cells. Activated Ras is limited to the leading edge of the cell. Ras stimulates PI3K, which triggers an intracellular PI(3,4,5)P_3_ gradient at the leading edge and F-actin polymerization (44).

Besides PI3K, cells have PI3K-independent pathways for F-actin polymerization and polarization. The TOR pathway was found to control cell polarity *via* Ras (49). TOR is a PI3K-related kinase with two protein complexes, TORC1 and TORC2. One of the principal functions of TORC2 is actin cytoskeleton rearrangement. The mechanisms by which TORC2 regulates actin reorganization is not well defined; although some studies have implicated Slm1 and Slm2, members of a family protein bind to PI(4,5)P_2_ and TORC2. Slm1 and Slm2 are activated by TORC2, and inactivation of Slm1 and Slm2 diminishes polarized cortical actin distribution (50, 51). These investigations from *Dictyostelium* demonstrate an important role of TOR complexes in chemotaxis *via* Ras. Therefore, Ras may regulate chemotaxis through PI3K and TOR pathways.

In summary, a model for feedback loop-mediated directional sensing and cell polarization has been proposed. “The chemoattractant locally stimulates Ras at the presumptive leading edge (site of the membrane closest to the chemoattractant source) where Ras locally activates PI3K. There is a local polymerization of F-actin at the presumptive leading edge which is dependent on Ras/PI3K and Ras/TORC2 pathways. Locally produced PI(3,4,5)P_3_ and F-actin-mediated Rac activation induces further F-actin polymerization and chemotactic movement of the cell” (44).

## Plants

### Mechanotransduction in Plants

The direction of growth of plants is primarily regulated by gravity (52). Plants can re-orientate themselves with respect to gravity. When a plant is turned in the gravitational field, the change in orientation is perceived by its organs. These organs respond to this stimulus by bending their extremity to recover the normal orientation with respect to gravity (53).

#### Movement of Amyloplasts in Response to Gravity

How do plants sense the direction of gravity? Two hypotheses have been proposed: the gravitational pressure model (54) and the starch-statolith hypothesis (55). The latter has been strongly supported by a variety of experimental approaches in several plants. Amyloplasts are defined as “non-pigmented organelles found in some plant cells. They are responsible for the synthesis and storage of starch granules, through the polymerization of glucose.” In most cases, statoliths are starch-accumulating amyloplasts that can sediment in the direction of gravity within specialized cells (statocytes) present in the columella and shoot endodermal cells. Columella endodermal cells have the endoplasmic reticulum (ER) on the peripheral side of the cells. A specialized form of ER, called nodal ER, is restricted to a peripheral layer underneath the plasma membrane (56). It has been suggested that the nodal ER could provide directional cues by identifying the orientation of the root in relation to gravity. In contrast, shoot endodermal cells are occupied by a large central vacuole. Genetic studies of *sg r* mutants have found that the contribution of vacuoles to gravity is by modifying amyloplast sedimentation (57). Therefore, columella and shoot endodermal cells seem to play key roles for gravity sensing on shoots and roots.

Past investigations have demonstrated an association between amyloplast movement and response to gravity (58, 59). Sack and Leopold (60) have described amyloplast movement in living statocytes. They observed that amyloplast sedimentation happened within the minimum time of continuous stimulus necessary to trigger gravity perception. Morita’s group (61) suggested that movement of amyloplasts toward the gravity vector is crucial to trigger gravity perception.

#### Cell Signaling Mechanisms after Amyloplast Sedimentation

Cytosolic ions mediate gravity signaling. Calcium is the most abundant ionic second messenger in plants. However, changes in cytoplasmic pH are also important regulators of cell function. It has been demonstrated, for example, that cytosolic calcium is transiently activated by gravity (62). They observed that after gravistimulation, there was an initial cytosolic calcium spike for 20–25 s, followed by a much longer shoulder that peaked around 90 s after the change in orientation of the plant. The investigators suggested that the “spike could be related to the early steps of gravisensing, whereas the shoulder could be related to the movement of the amyloplasts.”

Besides the ionic signaling, several proteins have been identified to participate in the cell signaling pathways related to gravitropism. The ARG1/RHG protein, for example, is mostly associated with cell membrane but a small portion also appears to be associated with the actin cytoskeleton (63). Functional ARG1/RHG protein is required for alkalinization in the root columella cells following gravistimulation. ARG1/RHG is present on several endomembrane systems, such as ER, the Golgi apparatus, and endosomes (64). Therefore, ARG1/RHG protein might function in the vesicular trafficking required for the alkalinization of columella cells.

#### Gravity Sensing and the Cytoskeleton

The cytoskeleton was the best candidate for transmitting the force exerted by gravisensors to the mechanoreceptors. Actin filaments in particular have been thought to play a major role in gravity sensing. It has been shown that in microgravity, the statoliths are not distributed at random in the root statocytes. When lentil seedlings are grown in a 1 × *g* centrifuge in space and then placed under microgravity conditions for various periods of time, the amyloplasts move in the direction of the proximal wall (65, 66). This movement is attributed to the presence of myosin around the amyloplasts (67). Therefore, the movements of the amyloplasts detected in microgravity demonstrate the interactions between amyloplasts and cytoskeleton. The role of cytoskeleton has been also demonstrated using the *arg1* mutant of *Arabidopsis* (63), which showed an altered response to gravity. However, other studies have shown conflicting results (68–70). Interestingly, organ curvature exceeding 90° was found in plants exposed to latrunculin-B to disrupt actin organization and in dominant actin mutant (69, 70), suggesting that actin cytoskeleton may be important to fine-tune the gravitropic response.

In summary, “gravisensing cells (statocytes) contain movable amyloplasts whose potential energy is apparently used to activate calcium channels by exerting tension on the actin network and/or pressure on the cytoskeleton elements lining the plasma membrane. The chain of events that follows remains to be further elucidated but includes pH changes in the cytosol and cell wall” (62). Transduction ends with relocation of the auxin, a signaling protein that regulates several plant functions, including cell elongation, which reorients the root tip in the direction of gravity (53).

## Animals

### Vestibular System

#### Mechanotransduction by Hair Cells

The vestibular system is important to maintain the body equilibrium under gravity. The detection of the gravitational force requires specific receptors. This is achieved by two otolithic organs of the vestibular system, the utricle and saccule (71). These gravity receptors utilize a layer of calcium carbonate (otoconia) lying over the sensory receptor areas. The shearing force produced by the otoconia displaced against the stereocilia of the sensory hair cells allows the detection of linear accelerations, and gravity (72).

“Hair bundles are the mechanosensitive organelles that transduce vestibular stimuli into electrical signals” (73). “Each vestibular end organ contains thousands of hair cells. Each hair cell has one bundle that contains 30–300 microvilli, or stereocilia. Stereocilia are composed of cores of hundreds of actin filaments covered by an extension of the cell membrane” (74). The actin filaments taper near their base and are anchored to the cell body in a thickened region known as the cuticular plate (75). This arrangement allows the stereocilia to hinge around the insertion into the cuticular plate (76). Furthermore, stereocilia are connected together, within a hair bundle and not to neighboring bundles (77). Therefore, stimulation of the hair bundle does not induce separation of the stereocilia but rather brings uniform bundle deflections (78). “The stereocilia are arranged in a staircase-like array with a single true cilium, the kinocilium, located adjacent to the top of the staircase. The kinocilium does not exhibit mechanosensitivity but provides a connection of the apex of the hair bundle to overlying structures and it may also serve to organize proper formation of the bundle during development” (79).

Transduction channels are located at stereociliary tips (80). Mechanotransduction is initiated when mechanical stimuli activate the stereocilia at the apical surface of the hair cells. Movement of the stereocilia induces the opening of cation channels with subsequent activation, changes in the membrane potential of the hair cells and release of neurotransmitters to send the information to the brain (81). The molecules that form the transduction channel have not been identified. However, recent studies in mice (82) have identified key roles for transmembrane channel-like 1 and -like 1 in hair cell mechanotransduction. The mechanisms by which tip links open the transduction channels are not know. However, two models for channel coupling and gating have been proposed, The Tethered-Channel Model and The Lateral Tension Model. In the first one, “the channel is connected to the cytoskeleton and to the tip link, so that forces in the tip link are propagated *via* protein-protein interactions to the channel.” In the lateral tension model, “the channel responds to tension in the stereocilia tip membrane. Increased tension in the tip link would then increase membrane tension and open channels” (83).

#### Vestibular Response to Microgravity

The maturation of gravity sensing is genetically programed and also depends on the exposure to the gravity (84, 85). The nervous system probably needs environmental experience to calibrate the gravity information during critical periods of development and several studies have shown the sensitivity of the organisms to the alteration of gravity during their development (86–90). The peripheral sensory organ adapts to the level of gravity by adjusting the mass of otoconia and the innervation of sensory epithelium. Understimulation, such as caused by microgravity, can delay the maturation of neural connections during the formation of the vestibular apparatus. “All together, these results provide further evidence that the gravistatic sensory system has a genetically controlled phase of development and a stimulus-controlled phase for fine-tuning synaptic terminals. Therefore, the level of gravity plays a critical role in fine-tuning of axons and is required for appropriate development of the projections from graviceptors to the brain and spinal cord” (72).

### Bone

#### Role of Osteocytes and Canaliculi in Bone Mechanotransduction

The skeleton has the capability to remodel in response to mechanical stimuli (91). Osteocytes comprise over 90% of all bone cells in adults. Osteocytes are considered the terminal differentiation stage of osteoblasts and are distributed all through the mineralized matrix, in particular the cortical bone. These cells are connected to each other and to cells on the surface *via* dendritic processes present inside small canals called canaliculi (92). Because of their distribution and extensive network connections, osteocytes are considered to play a central role in mechanotransduction of bone cells by sensing mechanical signals and regulating bone resorption and formation (93). Using live imaging of osteocytes expressing green fluorescent protein, Dallas and colleagues (94) demonstrated that osteocyte embedded in bone can extend and retract their dendritic processes and undergo cell body deformation. In addition, it has been suggested that osteocytes are activated by strain-driven motion of interstitial fluid through the lacunocanalicular porosity (95–97). *In vitro* studies have demonstrated that the flow of fluids through the canaliculi stimulates the release secondary messengers by osteocytes, including ATP, nitric oxide, Ca^2+^, and prostaglandins (98–101). These studies suggest that the movements of the fluids through the channels could be one of the main mechanical stimuli on osteocytes (11). The Wnt/β-catenin signaling pathway is an important modulator of bone mass and bone cell functions. This pathway is critical for osteoblasts differentiation, proliferation, and synthesis of bone matrix. The Wnt/β-catenin pathway seems to be important in osteocytes to transfer mechanical signals to cells located on the bone surface (102).

#### Effects of Microgravity on Bone Cells

Under microgravity conditions, bones no longer have to fight against Earth’s gravitational force during locomotion and less mechanical strain is applied to the skeletal system. Reduced bone strain is widely accepted to cause progressive bone loss under microgravity (103). There are four major types of bone cells: mesenchymal stem cells (MSCs), osteoblasts, osteocytes, and osteoclasts.

Mesenchymal stem cells are multipotent cells with high replication capacity and the potential to differentiate into different lineages of mesenchymal tissues, including bone, cartilage, fat, muscle, and marrow stroma (104). Mechanical forces can enhance the osteogenic differentiation of MSCs, while a lack of mechanical stimuli can induce adipogenesis (105). Past studies have demonstrated that simulated microgravity inhibits the osteogenic differentiation of MSCs and osteogenic precursor cells (106). Some authors attribute these changes to F-actin cytoskeletal disturbances and alterations of Rho GTPase activity (107).

Osteoblasts are derived from MSCs. Osteoblasts control bone formation and participate in the regulation of bone homeostasis (108). Microgravity inhibits the differentiation of osteoprogenitor cells into mature osteoblasts (107). Osteoblasts are considered responsible for the bone loss induced by microgravity, given their decreased proliferation, reduced differentiation, and decreased response to local factors under low gravity conditions. Previous investigations have shown that microgravity disrupts osteoblast microfilaments, resulting in defective bone formation (109, 110). In addition, low gravity can promote apoptosis by altering focal adhesions (111).

Osteocytes have been implicated as key effectors of microgravity-induced bone loss (112). Osteocyte apoptosis was found after a 2-week flight accompanied by an increasing the number of functionally active osteoclasts (113). Hypogravity compromises the cytoskeletal architecture and suppresses the gap junctions of osteocytes. Given the key role of gap junctions in cell-to-cell communication, these changes are going to compromise overall cell function (114).

Osteoclasts are multinucleated bone-resorbing cells (115). Osteoclast differentiation is enhanced in microgravity (116). Microarray analysis of osteoclasts in the modified rotating wall vessel RCCS showed an upregulation of genes involved in osteoclast differentiation (117). Several studies indicate that bone loss under microgravity is caused by a decrease in osteoblast function and an increase in osteoclastic activity (118, 119).

### Lung

#### The Lung in Gravity

The normal lung is markedly influenced by the presence of gravity. To better understand the effects of microgravity on the lung, it is important first to describe how gravity affects perfusion and ventilation in dependent versus non-dependent regions of the lung.

The zone model of pulmonary perfusion establishes that regional perfusion depends on the balance between pulmonary arterial pressure, pulmonary venous pressure, and alveolar pressure (120). Given the low perfusion pressures in the pulmonary circulation, hydrostatic pressure caused by gravity is important to determine pulmonary perfusion. Thus, in the lower parts of the lung, blood flow depends on difference between arterial and venous pressure. In contrast, at the top of the lung, and given the decrease of hydrostatic pressure by gravity, pulmonary pressures can fall below alveolar pressure and compromise blood flow. Therefore, there is a vertical gradient in blood flow in the different regions of the lung with blood flow greater in the dependent portions of the lungs.

The Slinky model of pulmonary ventilation (121) determines that the behavior of a spring is similar to the lung. If the spring is stretched, the loops at the top are more distant from each other than at the bottom. This is similar to alveolar size under resting conditions, with alveoli at the top being bigger than at the bottom. However, if the string is stretched further (to simulate inspiration), and due to the elastic recoil forces of the spring, the loops in the lower part of the spring are more distant apart from each other than in the upper part (and by comparison, ventilation is greater in the lower parts of the lung). If the effects of gravity are removed, this model would predict uniform alveolar size, ventilation and perfusion (122).

In summary, “gravity causes uneven ventilation in the lung through the deformation of lung tissue (Slinky effect), and uneven perfusion through a combination of the Slinky effect and the zone model of pulmonary perfusion” (122).

### The Lung in Microgravity

#### Lung Volumes and Expiratory Flows

Vital capacity showed an initial small reduction, but by day 4 in microgravity it returned to pre-flight values. In contrast, functional residual capacity fell in microgravity. This was probably caused by the elimination of the “push-down” effect of the abdominal contents. Unexpectedly, residual volume decreased in microgravity when compared to normal gravity. The likely explanation is that “under gravity, dependent regions of the lung reach their local residual volume before the entire lung does, and gas remains trapped in these regions. However, in microgravity, the uniform alveolar expansion permits a more uniform overall emptying of the lung and a lower total residual volume” (123).

#### Ventilation

Based on the Slinky model, one would expect that pulmonary ventilation in all regions of the lung should be identical under low gravity conditions. However, when single-breath tests were performed in spaceflight (124), ventilatory heterogeneity persisted to some degree. This could be perhaps explained by differences in regional lung shape. Another indicator of regional differences in ventilation is the presence of cardiogenic oscillations. Cardiogenic oscillations are generated by the expansion of the heart during diastole on the nearby lung, implying differences in ventilation between the lung close and distant to the heart. Contrary to expected, cardiogenic oscillations persisted near to 50% of the results under gravity. Therefore, these findings and the previous studies from the single-breath washout experiments demonstrate the presence of ventilatory heterogeneity in microgravity (125). To support these observations, other studies have also found that the lack of ventilatory uniformity during tidal breathing in the upright position was not primarily gravitational (126). Therefore, it looks like that the elastic properties of the lung have greater impact than gravity during tidal breathing.

#### Blood Flow

The effects of gravity on uneven distribution of pulmonary perfusion were tested under microgravity conditions using the hyperventilation-breathhold single-breath measurements. Under low gravity, the indicators of uneven pulmonary perfusion such as the size of cardiogenic oscillations in expired CO_2_ and the height of phase IV, were significantly reduced. The terminal change in expired CO_2_ was also reduced in microgravity, indicating more uniform blood flow between lung units that close and those that remain open at the end of expiration. A possible explanation of this observation is the disappearance of gravity-dependent distribution of blood flow. However, the presence of residual cardiogenic oscillations in expired CO_2_ implies a persisting non-homogenous distribution of perfusion even in the absence of gravity, probably in lung regions that are not within the same acinus (127).

#### Gas Exchange and Ventilation–Perfusion Matching

The principal change observed in microgravity was a slight decrease of alveolar ventilation (128). There was also a significant drop in resting tidal volume and simultaneous increase in respiratory rate (129). This was associated with a reduction in the physiological dead space. Given that the degree of heterogeneity of both ventilation and perfusion in the lung were reduced in microgravity, one would expect the V/Q to be reduced as well. However, the V/Q did not change when compared to normal gravity (130). The explanation is that under 1 × *g*, the areas of high ventilation had also high perfusion and vice versa. Therefore, “gravity imposes common effects on both ventilation and perfusion (the zone and Slinky models) serving to maintain a high gas exchange efficiency in the lung” (122).

#### Conclusion

The zone model of pulmonary blood flow and the Slinky model of lung deformation together provide a basis to understand how gravity affects lung function and how the lung changes in the absence of gravity. Under gravity, and despite the regional differences in ventilation and perfusion, the V/Q ratio remains constant and provides efficient gas exchange. In a weightless environment, and even with more uniform distribution of both ventilation and perfusion, the gas exchange seems to be no more efficient than in gravity. In addition, and contrary to other organs, the lung does not experience significant structural changes when gravity is removed and continues to function well [for a more comprehensive review, please see the excellent article by Prisk (122)].

#### Lung Gravisensors

Lung development is well known to be influenced by a highly active mechanical environment (131, 132). One possible interpretation of the link between physical force and development is the presence of gravisensors that have allowed organisms to adapt to gravity during evolution (133) and development (134). Previous studies have shown that lung cytoarchitecture is exquisitely sensitive to fluid distension in the womb, a process that would allow for sensing of gravitational forces on the developing conceptus (135, 136).

Parathyroid hormone-related protein (PTHrP) has been shown to be essential for the development and homeostatic regulation of lung and bone (137). PTHrP is expressed in a wide variety of tissues, including the uterus (138, 139), bladder (140), lung (141), and bone (142). During gestation, PTHrP levels increase in the distending uterus and then rapidly decrease when the uterus is evacuated at birth, suggesting that mechanical stretch causes the increased PTHrP expression. Moreover, studies by Torday’s research group have demonstrated that expression of PTHrP by the pulmonary epithelium is sensitive to stretch (136) and that production of PTHrP is necessary for the normal alveolar development (143).

Interestingly, prior investigations have demonstrated that PTHrP is a gravisensor in both lung and bone (144, 145). When either rat lung type II epithelial cells or human UMR 106 bone cells were suspended in a rotating wall vessel bioreactor, to simulate microgravity, they lost their PTHrP receptor-mediated signaling mechanism within 6–8 h (144). As a result, the cells established a new, lowered baseline of PTHrP expression. If the cells were put back into gravity, PTHrP signaling was restored within 24 h, suggesting that this was a reversible process. They concluded that PTHrP signaling pathway constitutes a mechanism by which these cells can sense gravity and alter their cellular and biochemical environment accordingly. In further support of the relevance of this gravisensing mechanism to microgravity adaptation, Torday’s group analyzed bones from rats flown in space on NASA Mission SL-2 (144, 145) Analysis of PTHrP receptor expression by the femurs and tibias of these animals revealed that PTHrP expression was 60% lower than in the bones from control ground-based rats. Interestingly, there were no differences in PTHrP expression by parietal bone from space-exposed versus ground-based animals, indicating that the effect of microgravity on PTHrP expression is specifically the result of the unweighting of the weight-bearing bones.

In conclusion, PTHrP is a mechanosensor found to be sensitive to changes in gravity environment. The link among physical force, development, and homeostasis support the hypothesis that there are gravisensors that have allowed organisms to physiologically adapt to gravity (137).

### Gene Expression Response to Microgravity

Several large-scale genome-wide studies have been performed in a variety of organisms such as human, rat, mouse, *Xenopus*, yeast, *Caenorhabditis elegans*, and *Drosophila* to understand the impact of gravity on living organisms (146).

#### Cells

Several studies have investigated the impact of low gravity on gene expression in isolated cells. Wang’s group (147) examined the effects of microgravity on WI-38 human fibroblasts. Their data show that spaceflight activates a group of genes involved in oxidative stress, DNA repair, and fatty acid oxidation. Clement et al. (148) investigated the effects of simulated microgravity on a keratinocyte cell line. They found that exposure to microgravity for a short period of time (3–4 days) had no persistent effect on gene expression. In contrast, longer exposure (9 or 10 days) exhibited substantial alterations in gene expression. Their results suggest that longer exposure to microgravity tends to have lasting effects on gene expression. Their experiments are also in agreement by studies performed by Lu et al. (149) demonstrating mutations in the content of albumin, globulin, and prolamine in rice seeds that were flown on a recoverable satellite for 15 days. These changes were stably inherited from several generations on earth. Altogether, these studies demonstrate that exposure to microgravity could have a long-lasting impact on gene expression at the cellular level.

#### Mammalians

Recent studies have investigated the impact of spaceflight on mammary transcriptome of late pregnant rats (150). Microarray analysis of the dams revealed that “alterations in gravity affected the expression of genes that regulate circadian clocks and activate mechanotransduction pathways. Changes in these systems may explain global gene expression changes in immune response, metabolism, and cell proliferation” (150).

The circadian system is important to synchronize the physiological oscillations with the external environment signals such as light, exercise, stress, and gravity. Several studies have demonstrated that changes in gravity significantly affect circadian rhythms of behavior, hormones, body temperature, and metabolism (151–156). Specifically for gravity, the vestibular system is the primary sensor that detects changes in gravitational force; impulses from these receptors are sent to the central circadian clock in the suprachiasmatic nuclei. When circadian rhythms are disrupted, an organism’s ability to respond appropriately to a physiological stressor is inhibited. Transcriptional signatures of microgravity rats showed changes in expression of multiple core clock and clock regulatory genes, indicating that microgravity affect the expression of genes that regulate circadian clocks (150).

#### Impact of Microgravity on Epigenetic Modifications

Epigenetics has been defined as the study of changes in gene function that are mitotically and/or meiotically heritable and that do not entail a change in DNA sequence. At least three systems including DNA methylation, histone modification and non-coding RNA-associated gene silencing are currently considered to initiate and sustain epigenetic modifications. Epigenetic changes, in particular DNA methylation, occur in response to various environmental factors. Epigenetic changes are inherited in somatic cells and provide a potential mechanism by which environmental effects on the epigenome can have long-term effects on gene expression (157). Epigenetic modifications are regulated by a family of enzymes, called DNA methyltransferases, transferring a methyl group to DNA. Expression of DNA methyltransferase 3α was decreased in microgravity when compared to 1 × *g* controls (150). DNMT3A is referred to as a *de novo* methyltransferase. DNMT3a can also mediate methylation-independent gene repression.

They also observed upregulation of Kruppel-like factor 4 (KLF4) in microgravity of mammary tissue. KLF4 is a member of the KLF family of transcription factors and regulates proliferation, differentiation, and tissue development, in part *via* chromatin modification. KLF4 has been found to participate in the reprograming of somatic cells into pluripotent stem cells *via* downregulation of DNA methylation on certain genes (158).

Members of the forkhead/winged helix transcription factor family were also affected by alterations in gravity in these investigations. FOXN3 was upregulated in mammary of spaceflight-exposed rats. FOX proteins participate in regulation of gene expression involved in cell growth, proliferation, differentiation, and longevity. They are able to control DNA methylation by binding condensed chromatin and recruiting other transcription factors and histone modification enzymes.

Other investigations, using simulated microgravity, have also observed epigenetic changes in human lymphoblastoid and lymphocyte cells (159, 160). However, none of these studies have demonstrated that these changes are heritable. Therefore, it is possible that microgravity only affects the transcription factors that are possibly linked with epigenetic effects on chromatin structure and cellular cytoskeletal physiology. In contrast, spaceflight induced both transient and heritable alterations in DNA methylation and gene expression in rice (161).

In summary, these studies show changes in genes that modify chromatin structure and methylation, suggesting that long-term adaptation to gravity might be mediated by epigenetic changes in DNA.

## Cytoskeleton

### Role of Cytoskeleton as Gravity Sensor

Mechanical forces, such as those due to gravity, play an important role in tissue development. However, the mechanisms by which living cells sense these mechanical signals and convert them into an intracellular response are still not well characterized. It is also controversial whether the forces induced by gravitational field on the cells are too small to induce any response (15). Despite this theoretical consideration, there are accumulative experimental data demonstrating that cultured cells are sensitive to gravity (12–14). Moreover, there is clear evidence that certain cells, such as the statoliths in plants and the otoliths of the inner ear, have developed specialized structure in response to gravity, indicating their ability to sense these mechanical signals.

The cytoskeleton provides shape and mechanical strength to cells. It consists of actin, microtubules, and intermediate filaments (162). The cytoskeleton is able to sense changes induced by gravity. However, to do so and promote changes at the cellular levels, this force has to be transmitted first to receptors on the cell membrane sensitive to mechanical signals and then to downstream signaling pathways that eventually will affect cell function.

### Tensegrity Model of Mechanotransduction

Past studies have suggested that “cells may sense mechanical stresses, including those due to gravity, through changes in the balance of forces that are transmitted across transmembrane adhesion receptors that link the cytoskeleton to the extracellular matrix and to other cells” (163–165). The mechanism by which these mechanical signals are converted into an intracellular response could be explained by a model, known as *tensegrity*, that describes how cell architecture detects and responds to stimuli (166).

In this model, the cellular response to stress differs depending on the level of pre-stress (pre-existing tension) in the cytoskeleton, and it involves all three cytoskeletal components as well as nuclear scaffolds (164, 165, 167, 168). The tensegrity model suggests that “cellular control lies in the balance of forces that are transmitted across cell surface adhesion receptors, through the cytoskeleton, and into the nucleus” (169). In this sense, we can think of integrins and other transmembrane adhesion receptors as mechanoreceptors. Past investigations have shown that alterations in the cellular force balance can activate not only specific signaling pathways at the focal adhesion–integrin complexes but also induce changes in gene expression (163, 170–173). Therefore, according to this model, cells in all tissues may sense changes in gravity *via* alterations in the balance of forces distributed between their adhesion receptors and the cytoskeleton, rather than through direct activation of any single receptor molecule.

“What does this mean for how gravity influences cell and tissue development? Local distortion in the cytoskeleton appears to be common to all mechanisms of cellular mechanotransduction. Certain specialized mechanosensory cells, such as otoliths and sterocilia, utilize highly dense microstructures to induce mechanical strain in the cytoskeleton in order to experience gravitational acceleration. Other non-specialized cells may also feel the pull of gravity as a result of cytoskeleton distortion” (9). According to Ingber’s model (9), the concept of a single gravity-specific receptor molecule and the idea that mechanical signals are transmitted equally at all points on the cell surface should be discarded. Instead, “gravity sensation should be viewed in the context of the structural complexity of living cells and tissues. In some cases the whole cell or even the whole tissue must be viewed as ‘the’ gravity sensor. In fact, gravity acting on the whole organism is a major contributor to pre-stress within individual tissues. When organisms are placed in microgravity, they experience an acute decrease in pre-stress on the macroscale which should produce corresponding changes in structure and mechanics at the cellular and molecular level” (166).

### Cytoskeletal Changes during Microgravity and Role of RhoGTPases

In adherent cells, microtubules have a radial organization and actin stress fibers are anchored to the cell membrane. At these sites, cells can attach to their extracellular matrix *via* focal adhesion complexes. Actin is also localized at the cell border. Intermediate filaments form a loose network (15).

Several studies have demonstrated that microtubules self-organization is influenced by the direction and magnitude of gravity (174, 175). Therefore, when cells are exposed to microgravity, microtubules experience a disruption of the radial pattern (176) showing a perinuclear clustering distribution (177). This specific localization could lead to a reduced rate of chromosome segregation during mitosis.

Actin stress fibers are also affected by gravitational forces. Under low gravity environment, actin stress fibers are reduced in number, length, and thickness (178, 179). Actin is often redistributed and has either a more perinuclear or more cortical localization (180, 181). Focal adhesion proteins no longer align well with the stress fibers; instead, they appear as bigger clusters without radial orientation in the cortical layer, resulting in reduced cell spreading (15). All these changes may be explained by a decrease of RhoA activity. The absence of gravity increases the G-actin form, which reduces cofilin phosphorylation, and decreases focal adhesions and stress fibers formation (182). Microgravity has also an impact on intermediate filaments, which after 12 min in microgravity form clusters preferentially distributed around the nucleus (183).

The RhoGTPases, RhoA, Rac1, and Cdc42, are members of the Ras superfamily of small GTP-binding proteins that play key roles in cytoskeletal dynamics (184, 185). RhoA, for example, regulates focal adhesion assembly, stress fiber formation, and intracellular tension. Rac1 primarily controls actin assembly and formation of lamellipodia to ensure cell migration. RhoGTPases play an important role integrating mechanical and biochemical signals. Therefore, they seem to be at the forefront in cell adaptation to microgravity. According to Louis et al. (186), on 1 × *g*, cell tension is sensed through the cytoskeleton *via* microtubules, intermediate filaments, and actin stress fibers associated with focal adhesions within the extracellular matrix. These elements are controlled by GTPases RhoA and Rac1. During short-term exposure to microgravity, RhoA is inhibited to allow reduction in cell tension to adapt to the new mechanical environment. At the same time, Rac1 is activated to control peripheral actin polymerization. All these events lead rapidly to a rounder cell shape with disorganization of microtubules, stress fibers, intermediate filaments, and focal adhesions. Transcription may be also altered as nucleus shape is changed.

## Conclusion

Understanding how living organisms have adapted to gravity is very complex, and the mechanisms responsible for this adaptation remain controversial. However, several of the studies reviewed provide some clues. For example, in an excellent review, Jamon (72) shows that the vestibular response to microgravity is not only genetically programed but also depends on the exposure to gravity. “The gravistatic sensory system has a genetically controlled phase of development and a stimulus-controlled phase for fine-tuning synaptic terminals. Therefore, the level of gravity plays a critical role in fine-tuning of axons and is required for appropriate development of the projections from graviceptors to the brain and spinal cord” (72).

Moreover, several large-scale genomic-wide studies have been performed in various organisms to understand the impact of gravity on living organisms (146). These investigations have shown that cell proliferation, growth, differentiation, signaling, shape, and gene expression are all altered by changes in gravity. Interestingly, exposure to microgravity for a short period of time had no persistent effect on gene expression. In contrast, longer exposure can induce mutations in some genes that are stably inherited for several generations. Another important clue from mammalian experiments is that long-term adaptation to gravity may be also mediated by epigenetic changes in DNA.

It is now widely accepted that the cytoskeleton plays a role in sensing changes in gravity. However, the mechanisms by which living cells sense these signals and convert them into a biochemical response are still not well characterized. It is also controversial whether the forces induced by gravitational field on the cells are too small to induce any response (15). Given that gravity represents a modification in mechanical stresses encounter by the cells, the tensegrity model of cytoskeletal architecture (9) provides an excellent paradigm to explain how changes in the balance of forces, which are transmitted across transmembrane receptors and cytoskeleton, can influence intracellular signaling pathways and changes in gene expression.

## Author Contributions

TN and JS-E have contributed to the revision of this article. JS-E has supervised and revised the final version.

## Conflict of Interest Statement

The authors declare that the research was conducted in the absence of any commercial or financial relationships that could be construed as a potential conflict of interest.

## References

[1] Ditsche P Summers AP. Aquatic versus terrestrial attachment: water makes a difference. Beilstein J Nanotechnol (2014) 5:2424–39. 10.3762/bjnano.5.252 25671138 PMC4311720

[2] Morey-Holton ER The impact of gravity on life. In: Rothschild LJ Lister AM, editors. Evolution on Plant Earth: The Impact of the Physical Environment. San Diego, CA: Academic Press, An Imprint of Elsevier (2003). p. 143–60.

[3] Pravin S Mellon D Jr Berger EJ Reidenbach MA. Effects of sensilla morphology on mechanosensory sensitivity in the crayfish. Bioinspir Biomim (2015) 10:036006. 10.1088/1748-3190/10/3/036006 25909394

[4] McHenry MJ Strother JA Van Netten SM. Mechanical filtering by the boundary layer and fluid-structure interaction in the superficial neuromast of the fish lateral line system. J Comp Physiol A Neuroethol Sens Neural Behav Physiol (2008) 194:795–810. 10.1007/s00359-008-0350-2 18709377

[5] Lillywhite HB Snakes, blood circulation and gravity. Sci Am (1988) 256:92–8. 10.1038/scientificamerican1288-92

[6] Thewissen JG Williams EM The early evolution of Cetacea (whales, dolphins, and porpoises). Annu Rev Ecol Syst (2002) 33:73–90. 10.1146/annurev.ecolsys.33.020602.095426

[7] Nummela S Thewissen JG Bajpai S Hussain ST Kumar K. Eocene evolution of whale hearing. Nature (2004) 430:776–8. 10.1038/nature02720 15306808

[8] Nummela S Thewissen JG Bajpai S Hussain T Kumar K. Sound transmission in archaic and modern whales: anatomical adaptations for underwater hearing. Anat Rec (Hoboken) (2007) 290:716–33. 10.1002/ar.20528 17516434

[9] Ingber D. How cells (might) sense microgravity. FASEB J (1999) 13(Suppl):S3–15. 10352140 10.1096/fasebj.13.9001.s3

[10] Vernikos J. Human physiology in space. Bioessays (1996) 18:1029–37. 10.1002/bies.950181215 8976162

[11] Arfat Y Xiao WZ Iftikhar S Zhao F Li DJ Sun YL Physiological effects of microgravity on bone cells. Calcif Tissue Int (2014) 94:569–79. 10.1007/s00223-014-9851-x 24687524

[12] Cogoli A Cogoli-Greuter M. Activation and proliferation of lymphocytes and other mammalian cells in microgravity. Adv Space Biol Med (1997) 6:33–79. 10.1016/S1569-2574(08)60077-5 9048133

[13] Nichols HL Zhang N Wen X. Proteomics and genomics of microgravity. Physiol Genomics (2006) 26:163–71. 10.1152/physiolgenomics.00323.2005 16705019

[14] Grimm D Wise P Lebert M Richter P Baatout S. How and why does the proteome respond to microgravity? Expert Rev Proteomics (2011) 8:13–27. 10.1586/epr.10.105 21329425

[15] Vorselen D Roos WH Mackintosh FC Wuite GJ Van Loon JJ. The role of the cytoskeleton in sensing changes in gravity by nonspecialized cells. FASEB J (2014) 28:536–47. 10.1096/fj.13-236356 24249634

[16] Lesnyak A Sonnenfeld G Avery L Konstantinova I Rykova M Meshkov D Effect of SLS-2 spaceflight on immunologic parameters of rats. J Appl Physiol (1985) (1996) 81:178–82. 8828661 10.1152/jappl.1996.81.1.178

[17] Taylor GR Konstantinova I Sonnenfeld G Jennings R. Changes in the immune system during and after spaceflight. Adv Space Biol Med (1997) 6:1–32. 10.1016/S1569-2574(08)60076-3 9048132

[18] Nickerson CA Ott CM Wilson JW Ramamurthy R Pierson DL. Microbial responses to microgravity and other low-shear environments. Microbiol Mol Biol Rev (2004) 68:345–61. 10.1128/MMBR.68.2.345-361.2004 15187188 PMC419922

[19] Klaus D Simske S Todd P Stodieck L. Investigation of space flight effects on *Escherichia coli* and a proposed model of underlying physical mechanisms. Microbiology (1997) 143(Pt 2):449–55. 10.1099/00221287-143-2-449 9043122

[20] Tixador R Richoilley G Gasset G Templier J Bes JC Moatti N Study of minimal inhibitory concentration of antibiotics on bacteria cultivated in vitro in space (Cytos 2 experiment). Aviat Space Environ Med (1985) 56:748–51. 3899095

[21] Nickerson CA Ott CM Mister SJ Morrow BJ Burns-Keliher L Pierson DL. Microgravity as a novel environmental signal affecting *Salmonella enterica* serovar Typhimurium virulence. Infect Immun (2000) 68:3147–52. 10.1128/IAI.68.6.3147-3152.2000 10816456 PMC97548

[22] Wilson JW Ott CM Ramamurthy R Porwollik S Mcclelland M Pierson DL Low-Shear modeled microgravity alters the *Salmonella enterica* serovar typhimurium stress response in an RpoS-independent manner. Appl Environ Microbiol (2002) 68:5408–16. 10.1128/AEM.68.11.5408-5416.2002 12406731 PMC129924

[23] Wilson JW Ramamurthy R Porwollik S Mcclelland M Hammond T Allen P Microarray analysis identifies *Salmonella* genes belonging to the low-shear modeled microgravity regulon. Proc Natl Acad Sci U S A (2002) 99:13807–12. 10.1073/pnas.212387899 12370447 PMC129779

[24] Nickerson CA Ott CM Wilson JW Ramamurthy R LeBlanc CL Höner Zu Bentrup K Low-shear modeled microgravity: a global environmental regulatory signal affecting bacterial gene expression, physiology, and pathogenesis. J Microbiol Methods (2003) 54:1–11. 10.1016/S0167-7012(03)00018-6 12732416

[25] Blount P Moe PC. Bacterial mechanosensitive channels: integrating physiology, structure and function. Trends Microbiol (1999) 7:420–4. 10.1016/S0966-842X(99)01594-2 10498951

[26] Poolman B Blount P Folgering JH Friesen RH Moe PC Van Der Heide T. How do membrane proteins sense water stress? Mol Microbiol (2002) 44:889–902. 10.1046/j.1365-2958.2002.02894.x 12010487

[27] Thomas WE Trintchina E Forero M Vogel V Sokurenko EV. Bacterial adhesion to target cells enhanced by shear force. Cell (2002) 109:913–23. 10.1016/S0092-8674(02)00796-1 12110187

[28] Blount P. Molecular mechanisms of mechanosensation: big lessons from small cells. Neuron (2003) 37:731–4. 10.1016/S0896-6273(03)00122-3 12628164

[29] Thevenet D D’ari R Bouloc P. The SIGNAL experiment in BIORACK: *Escherichia coli* in microgravity. J Biotechnol (1996) 47:89–97. 10.1016/0168-1656(96)01384-3 8987563

[30] Brooks DE Trust TJ Interactions of erythrocytes with bacteria under shear. Ann N Y Acad Sci (1983) 416:319–31. 10.1111/j.1749-6632.1983.tb35196.x 6145382

[31] Isberg RR Barnes P. Dancing with the host; flow-dependent bacterial adhesion. Cell (2002) 110:1–4. 10.1016/S0092-8674(02)00821-8 12150990

[32] Purevdorj-Gage B Sheehan KB Hyman LE. Effects of low-shear modeled microgravity on cell function, gene expression, and phenotype in *Saccharomyces cerevisiae*. Appl Environ Microbiol (2006) 72:4569–75. 10.1128/AEM.03050-05 16820445 PMC1489333

[33] Sheehan KB Mcinnerney K Purevdorj-Gage B Altenburg SD Hyman LE. Yeast genomic expression patterns in response to low-shear modeled microgravity. BMC Genomics (2007) 8:3. 10.1186/1471-2164-8-3 17201921 PMC1774566

[34] Lynch SV Brodie EL Matin A. Role and regulation of sigma S in general resistance conferred by low-shear simulated microgravity in *Escherichia coli*. J Bacteriol (2004) 186:8207–12. 10.1128/JB.186.24.8207-8212.2004 15576768 PMC532419

[35] Watson A Mata J Bahler J Carr A Humphrey T. Global gene expression responses of fission yeast to ionizing radiation. Mol Biol Cell (2004) 15:851–60. 10.1091/mbc.E03-08-0569 14668484 PMC329398

[36] Kang PJ Sanson A Lee B Park HO. A GDP/GTP exchange factor involved in linking a spatial landmark to cell polarity. Science (2001) 292:1376–8. 10.1126/science.1060360 11313501 PMC4386611

[37] Colman-Lerner A Chin TE Brent R. Yeast Cbk1 and Mob2 activate daughter-specific genetic programs to induce asymmetric cell fates. Cell (2001) 107:739–50. 10.1016/S0092-8674(01)00596-7 11747810

[38] Amon A Mother and daughter are doing fine: asymmetric cell division in yeast. Cell (1996) 84:651–4. 10.1016/S0092-8674(00)81041-7 8625401

[39] Chant J Cell polarity in yeast. Annu Rev Cell Dev Biol (1999) 15:365–91. 10.1146/annurev.cellbio.15.1.365 10611966

[40] Saito H Tatebayashi K. Regulation of the osmoregulatory HOG MAPK cascade in yeast. J Biochem (2004) 136:267–72. 10.1093/jb/mvh135 15598881

[41] Pollard TD Borisy GG. Cellular motility driven by assembly and disassembly of actin filaments. Cell (2003) 112:453–65. 10.1016/S0092-8674(03)00120-X 12600310

[42] Friedl P. Prespecification and plasticity: shifting mechanisms of cell migration. Curr Opin Cell Biol (2004) 16:14–23. 10.1016/j.ceb.2003.11.001 15037300

[43] Parent CA. Making all the right moves: chemotaxis in neutrophils and *Dictyostelium*. Curr Opin Cell Biol (2004) 16:4–13. 10.1016/j.ceb.2003.11.008 15037299

[44] Sasaki AT Firtel RA Regulation of chemotaxis by the orchestrated activation of Ras, PI3K, and TOR. Eur J Cell Biol (2006) 85:873–95. 10.1016/j.ejcb.2006.04.007 16740339

[45] Tuxworth RI Cheetham JL Machesky LM Spiegelmann GB Weeks G Insall RH. *Dictyostelium* RasG is required for normal motility and cytokinesis, but not growth. J Cell Biol (1997) 138:605–14. 10.1083/jcb.138.3.605 9245789 PMC2141629

[46] Wilkins A Khosla M Fraser DJ Spiegelman GB Fisher PR Weeks G *Dictyostelium* RasD is required for normal phototaxis, but not differentiation. Genes Dev (2000) 14:1407–13. 10.1101/gad.14.11.1407 10837033 PMC316659

[47] Insall RH Borleis J Devreotes PN. The aimless RasGEF is required for processing of chemotactic signals through G-protein-coupled receptors in *Dictyostelium*. Curr Biol (1996) 6:719–29. 10.1016/S0960-9822(09)00453-9 8793298

[48] Sasaki AT Chun C Takeda K Firtel RA. Localized Ras signaling at the leading edge regulates PI3K, cell polarity, and directional cell movement. J Cell Biol (2004) 167:505–18. 10.1083/jcb.200406177 15534002 PMC2172490

[49] Lee S Comer FI Sasaki A Mcleod IX Duong Y Okumura K TOR complex 2 integrates cell movement during chemotaxis and signal relay in *Dictyostelium*. Mol Biol Cell (2005) 16:4572–83. 10.1091/mbc.E05-04-0342 16079174 PMC1237065

[50] Hresko RC Mueckler M. mTOR.RICTOR is the Ser473 kinase for Akt/protein kinase B in 3T3-L1 adipocytes. J Biol Chem (2005) 280:40406–16. 10.1074/jbc.M508361200 16221682

[51] Sarbassov DD Guertin DA Ali SM Sabatini DM. Phosphorylation and regulation of Akt/PKB by the rictor-mTOR complex. Science (2005) 307:1098–101. 10.1126/science.1106148 15718470

[52] Rosen E Chen R Masson PH. Root gravitropism: a complex response to a simple stimulus? Trends Plant Sci (1999) 4:407–12. 10.1016/S1360-1385(99)01472-7 10498965

[53] Perbal G Driss-Ecole D. Mechanotransduction in gravisensing cells. Trends Plant Sci (2003) 8:498–504. 10.1016/j.tplants.2003.09.005 14557047

[54] Staves MP. Cytoplasmic streaming and gravity sensing in *Chara* internodal cells. Planta (1997) 203:S79–84. 10.1007/PL00008119 11540332

[55] Sack FD. Plastids and gravitropic sensing. Planta (1997) 203:S63–8. 10.1007/PL00008116 11540330

[56] Zheng HQ Staehelin LA. Nodal endoplasmic reticulum, a specialized form of endoplasmic reticulum found in gravity-sensing root tip columella cells. Plant Physiol (2001) 125:252–65. 10.1104/pp.125.1.252 11154334 PMC61007

[57] Yano D Sato M Saito C Sato MH Morita MT Tasaka M. A SNARE complex containing SGR3/AtVAM3 and ZIG/VTI11 in gravity-sensing cells is important for *Arabidopsis* shoot gravitropism. Proc Natl Acad Sci U S A (2003) 100:8589–94. 10.1073/pnas.1430749100 12815100 PMC166273

[58] Kuznetsov OA Hasenstein KH. Magnetophoretic induction of curvature in coleoptiles and hypocotyls. J Exp Bot (1997) 48:1951–7. 10.1093/jexbot/48.316.1951 11541075

[59] Fitzelle KJ Kiss JZ. Restoration of gravitropic sensitivity in starch-deficient mutants of *Arabidopsis* by hypergravity. J Exp Bot (2001) 52:265–75. 10.1093/jexbot/52.355.265 11283171

[60] Sack FD Leopold AC Cytoplasmic streaming affects gravity-induced amyloplast sedimentation in maize coleoptiles. Planta (1985) 164:56–62. 10.1007/BF00391025 11540857

[61] Morita MT Tasaka M. Gravity sensing and signaling. Curr Opin Plant Biol (2004) 7:712–8. 10.1016/j.pbi.2004.09.001 15491921

[62] Plieth C Trewavas AJ. Reorientation of seedlings in the earth’s gravitational field induces cytosolic calcium transients. Plant Physiol (2002) 129:786–96. 10.1104/pp.011007 12068119 PMC161701

[63] Sedbrook JC Chen R Masson PH. ARG1 (altered response to gravity) encodes a DnaJ-like protein that potentially interacts with the cytoskeleton. Proc Natl Acad Sci U S A (1999) 96:1140–5. 10.1073/pnas.96.3.1140 9927707 PMC15364

[64] Boonsirichai K Sedbrook JC Chen R Gilroy S Masson PH. Altered response to gravity is a peripheral membrane protein that modulates gravity-induced cytoplasmic alkalinization and lateral auxin transport in plant statocytes. Plant Cell (2003) 15:2612–25. 10.1105/tpc.015560 14507996 PMC280565

[65] Volkmann D Baluska F Lichtscheidl I Driss-Ecole D Perbal G. Statoliths motions in gravity-perceiving plant cells: does actomyosin counteract gravity? FASEB J (1999) 13(Suppl):S143–7. 10352156 10.1096/fasebj.13.9001.s143

[66] Driss-Ecole D Jeune B Prouteau M Julianus P Perbal G. Lentil root statoliths reach a stable state in microgravity. Planta (2000) 211:396–405. 10.1007/s004250000298 10987559

[67] Baluska F Hasenstein KH. Root cytoskeleton: its role in perception of and response to gravity. Planta (1997) 203:S69–78. 10.1007/PL00008117 11540335

[68] Blancaflor EB. The cytoskeleton and gravitropism in higher plants. J Plant Growth Regul (2002) 21:120–36. 10.1007/s003440010041 12024227

[69] Yamamoto K Kiss JZ. Disruption of the actin cytoskeleton results in the promotion of gravitropism in inflorescence stems and hypocotyls of *Arabidopsis*. Plant Physiol (2002) 128:669–81. 10.1104/pp.010804 11842170 PMC148928

[70] Hou G Mohamalawari DR Blancaflor EB. Enhanced gravitropism of roots with a disrupted cap actin cytoskeleton. Plant Physiol (2003) 131:1360–73. 10.1104/pp.014423 12644685 PMC166895

[71] Colclasure JC Holt JR. Transduction and adaptation in sensory hair cells of the mammalian vestibular system. Gravit Space Biol Bull (2003) 16(2):61–70. 12959133

[72] Jamon M. The development of vestibular system and related functions in mammals: impact of gravity. Front Integr Neurosci (2014) 8:11. 10.3389/fnint.2014.00011 24570658 PMC3916785

[73] Vollrath MA Kwan KY Corey DP. The micromachinery of mechanotransduction in hair cells. Annu Rev Neurosci (2007) 30:339–65. 10.1146/annurev.neuro.29.051605.112917 17428178 PMC2865174

[74] Tilney LG Derosier DJ Mulroy MJ. The organization of actin filaments in the stereocilia of cochlear hair cells. J Cell Biol (1980) 86:244–59. 10.1083/jcb.86.1.244 6893452 PMC2110658

[75] Jacobs RA Hudspeth AJ Ultrastructural correlates of mechanoelectrical transduction in hair cells of the bullfrog’s internal ear. Cold Spring Harb Symp Quant Biol (1990) 55:547–61. 10.1101/SQB.1990.055.01.053 1983446

[76] Bashtanov ME Goodyear RJ Richardson GP Russell IJ. The mechanical properties of chick (*Gallus domesticus*) sensory hair bundles: relative contributions of structures sensitive to calcium chelation and subtilisin treatment. J Physiol (2004) 559:287–99. 10.1113/jphysiol.2004.065565 15218063 PMC1665060

[77] Goodyear R Richardson G. The ankle-link antigen: an epitope sensitive to calcium chelation associated with the hair-cell surface and the calycal processes of photoreceptors. J Neurosci (1999) 19:3761–72. 10234008 10.1523/JNEUROSCI.19-10-03761.1999 PMC6782689

[78] Duncan RK Eisen MD Saunders JC. Distal separation of chick cochlear hair cell stereocilia: analysis of contact-constraint models. Hear Res (1999) 127:22–30. 10.1016/S0378-5955(98)00168-3 9925013

[79] Hudspeth AJ Jacobs R. Stereocilia mediate transduction in vertebrate hair cells (auditory system/cilium/vestibular system). Proc Natl Acad Sci U S A (1979) 76:1506–9. 10.1073/pnas.76.3.1506 312502 PMC383283

[80] Beurg M Nam JH Crawford A Fettiplace R. The actions of calcium on hair bundle mechanics in mammalian cochlear hair cells. Biophys J (2008) 94:2639–53. 10.1529/biophysj.107.123257 18178649 PMC2267152

[81] Dallos P Santos-Sacchi J Flock A. Intracellular recordings from cochlear outer hair cells. Science (1982) 218:582–4. 10.1126/science.7123260 7123260

[82] Kawashima Y Geleoc GS Kurima K Labay V Lelli A Asai Y Mechanotransduction in mouse inner ear hair cells requires transmembrane channel-like genes. J Clin Invest (2011) 121:4796–809. 10.1172/JCI60405 22105175 PMC3223072

[83] Gillespie PG Muller U. Mechanotransduction by hair cells: models, molecules, and mechanisms. Cell (2009) 139:33–44. 10.1016/j.cell.2009.09.010 19804752 PMC2888516

[84] Fritzsch B Maklad A Bruce LL Crapon De Caprona MD. Development of the ear and of connections between the ear and the brain: is there a role for gravity? Adv Space Res (2001) 28:595–600. 10.1016/S0273-1177(01)00387-8 11803959

[85] Fritzsch B. Molecular developmental neurobiology of formation, guidance and survival of primary vestibular neurons. Adv Space Res (2003) 32:1495–500. 10.1016/S0273-1177(03)90387-5 15000110

[86] Walton KD Lieberman D Llinas A Begin M Llinas RR. Identification of a critical period for motor development in neonatal rats. Neuroscience (1992) 51:763–7. 10.1016/0306-4522(92)90517-6 1488121

[87] Moody SA Golden C Developmental biology research in space: issues and directions in the era of the international space station. Dev Biol (2000) 228:1–5. 10.1006/dbio.2000.9907 11087621

[88] Ronca AE Fritzsch B Alberts JR Bruce LL. Effects of microgravity on vestibular development and function in rats: genetics and environment. Korean J Biol Sci (2000) 4:215–21. 10.1080/12265071.2000.9647547 12760372

[89] Wubbels RJ Van Marle J Sondag HN De Jong HA. Effects of hypergravity on the morphological properties of the vestibular sensory epithelium. II. Life-long exposure of rats including embryogenesis. Brain Res Bull (2002) 58:575–80. 10.1016/S0361-9230(02)00828-6 12372561

[90] Ronca AE Fritzsch B Bruce LL Alberts JR. Orbital spaceflight during pregnancy shapes function of mammalian vestibular system. Behav Neurosci (2008) 122:224–32. 10.1037/0735-7044.122.1.224 18298265 PMC2610337

[91] Burr DB Robling AG Turner CH Effects of biomechanical stress on bones in animals. Bone (2002) 30:781–6. 10.1016/S8756-3282(02)00707-X 11996920

[92] Bonewald L Osteocytes. 3rd ed In: Marcus DFR Nelson D Rosen C, editors. Osteoporosis. Burlington, MA: Elsevier Academic Press (2007).

[93] Lanyon LE. Osteocytes, strain detection, bone modeling and remodeling. Calcif Tissue Int (1993) 53(Suppl 1):S102–6; discussion S106–107. 10.1007/BF01673415 8275362

[94] Dallas SL Veno PA Rosser JL Barragan-Adjemian C Rowe DW Kalajzic I Time lapse imaging techniques for comparison of mineralization dynamics in primary murine osteoblasts and the late osteoblast/early osteocyte-like cell line MLO-A5. Cells Tissues Organs (2009) 189:6–11. 10.1159/000151745 18728354 PMC2824180

[95] Burger EH Klein-Nulend J Mechanotransduction in bone – role of the lacuno-canalicular network. FASEB J (1999) 13(Suppl):S101–12. 10352151

[96] Riddle RC Donahue HJ. From streaming-potentials to shear stress: 25 years of bone cell mechanotransduction. J Orthop Res (2009) 27:143–9. 10.1002/jor.20723 18683882

[97] Temiyasathit S Jacobs CR. Osteocyte primary cilium and its role in bone mechanotransduction. Ann N Y Acad Sci (2010) 1192:422–8. 10.1111/j.1749-6632.2009.05243.x 20392268 PMC3999479

[98] Klein-Nulend J Semeins CM Ajubi NE Nijweide PJ Burger EH Pulsating fluid flow increases nitric oxide (NO) synthesis by osteocytes but not periosteal fibroblasts – correlation with prostaglandin upregulation. Biochem Biophys Res Commun (1995) 217:640–8. 10.1006/bbrc.1995.2822 7503746

[99] Cherian PP Siller-Jackson AJ Gu S Wang X Bonewald LF Sprague E Mechanical strain opens connexin 43 hemichannels in osteocytes: a novel mechanism for the release of prostaglandin. Mol Biol Cell (2005) 16:3100–6. 10.1091/mbc.E04-10-0912 15843434 PMC1165395

[100] Genetos DC Kephart CJ Zhang Y Yellowley CE Donahue HJ. Oscillating fluid flow activation of gap junction hemichannels induces ATP release from MLO-Y4 osteocytes. J Cell Physiol (2007) 212:207–14. 10.1002/jcp.21021 17301958 PMC2929812

[101] Lu XL Huo B Park M Guo XE. Calcium response in osteocytic networks under steady and oscillatory fluid flow. Bone (2012) 51:466–73. 10.1016/j.bone.2012.05.021 22750013 PMC3412915

[102] Bonewald LF Johnson ML. Osteocytes, mechanosensing and Wnt signaling. Bone (2008) 42:606–15. 10.1016/j.bone.2007.12.224 18280232 PMC2349095

[103] Hughes-Fulford M. Function of the cytoskeleton in gravisensing during spaceflight. Adv Space Res (2003) 32:1585–93. 10.1016/S0273-1177(03)90399-1 15002415

[104] Pittenger MF Mackay AM Beck SC Jaiswal RK Douglas R Mosca JD Multilineage potential of adult human mesenchymal stem cells. Science (1999) 284:143–7. 10.1126/science.284.5411.143 10102814

[105] Khayat G Rosenzweig DH Quinn TM. Low frequency mechanical stimulation inhibits adipogenic differentiation of C3H10T1/2 mesenchymal stem cells. Differentiation (2012) 83:179–84. 10.1016/j.diff.2011.12.004 22381625

[106] Huang Y Dai ZQ Ling SK Zhang HY Wan YM Li YH. Gravity, a regulation factor in the differentiation of rat bone marrow mesenchymal stem cells. J Biomed Sci (2009) 16:87. 10.1186/1423-0127-16-87 19772591 PMC2754420

[107] Zayzafoon M Gathings WE Mcdonald JM. Modeled microgravity inhibits osteogenic differentiation of human mesenchymal stem cells and increases adipogenesis. Endocrinology (2004) 145:2421–32. 10.1210/en.2003-1156 14749352

[108] Papachroni KK Karatzas DN Papavassiliou KA Basdra EK Papavassiliou AG. Mechanotransduction in osteoblast regulation and bone disease. Trends Mol Med (2009) 15:208–16. 10.1016/j.molmed.2009.03.001 19362057

[109] Dai Z Li Y Ding BS Zhang X Tan Y Wan YM Actin microfilaments participate in the regulation of the COLIAI promotor activity in ROS17/2.8 cells under simulated microgravity. Adv Space Res (2006) 38:1159–67. 10.1016/j.asr.2006.02.060

[110] Kumei Y Morita S Katano H Akiyama H Hirano M Oyha K Microgravity signal ensnarls cell adhesion, cytoskeleton, and matrix proteins of rat osteoblasts: osteopontin, CD44, osteonectin, and alpha-tubulin. Ann N Y Acad Sci (2006) 1090:311–7. 10.1196/annals.1378.034 17384275

[111] Nabavi N Khandani A Camirand A Harrison RE. Effects of microgravity on osteoclast bone resorption and osteoblast cytoskeletal organization and adhesion. Bone (2011) 49:965–74. 10.1016/j.bone.2011.07.036 21839189

[112] Lin C Jiang X Dai Z Guo X Weng T Wang J Sclerostin mediates bone response to mechanical unloading through antagonizing Wnt/beta-catenin signaling. J Bone Miner Res (2009) 24:1651–61. 10.1359/jbmr.090411 19419300

[113] Rodionova NV Polkovenko OV Oganov VS. Interactions of cells in zones of bone resorption under microgravity and hypokinesia. J Gravit Physiol (2004) 11(2):147–51. 16237820

[114] Di SM Qian AR Qu LN Zhang W Wang Z Ding C Graviresponses of osteocytes under altered gravity. Adv Space Res (2011) 48:1161–6. 10.1016/j.asr.2011.05.030

[115] Segovia-Silvestre T Neutzsky-Wulff AV Sorensen MG Christiansen C Bollerslev J Karsdal MA Advances in osteoclast biology resulting from the study of osteopetrotic mutations. Hum Genet (2009) 124:561–77. 10.1007/s00439-008-0583-8 18987890

[116] Tamma R Colaianni G Camerino C Di Benedetto A Greco G Strippoli M Microgravity during spaceflight directly affects in vitro osteoclastogenesis and bone resorption. FASEB J (2009) 23:2549–54. 10.1096/fj.08-127951 19329761

[117] Sambandam Y Blanchard JJ Daughtridge G Kolb RJ Shanmugarajan S Pandruvada SN Microarray profile of gene expression during osteoclast differentiation in modelled microgravity. J Cell Biochem (2010) 111:1179–87. 10.1002/jcb.22840 20717918

[118] Rucci N Rufo A Alamanou M Teti A. Modeled microgravity stimulates osteoclastogenesis and bone resorption by increasing osteoblast RANKL/OPG ratio. J Cell Biochem (2007) 100:464–73. 10.1002/jcb.21059 16927271

[119] Saxena R Pan G Dohm ED Mcdonald JM. Modeled microgravity and hindlimb unloading sensitize osteoclast precursors to RANKL-mediated osteoclastogenesis. J Bone Miner Metab (2011) 29:111–22. 10.1007/s00774-010-0201-4 20589403 PMC3000895

[120] West JB Dollery CT Naimark A Distribution of blood flow in isolated lung; relation to vascular and alveolar pressures. J Appl Physiol (1964) 19:713–24. 14195584 10.1152/jappl.1964.19.4.713

[121] Hopkins SR Henderson AC Levin DL Yamada K Arai T Buxton RB Vertical gradients in regional lung density and perfusion in the supine human lung: the Slinky effect. J Appl Physiol (1985) (2007) 103:240–8. 10.1152/japplphysiol.01289.2006 17395757 PMC2399899

[122] Prisk GK. Microgravity and the respiratory system. Eur Respir J (2014) 43:1459–71. 10.1183/09031936.00001414 24603820

[123] Elliott AR Prisk GK Guy HJ West JB. Lung volumes during sustained microgravity on Spacelab SLS-1. J Appl Physiol (1985) (1994) 77:2005–14. 7836229 10.1152/jappl.1994.77.4.2005

[124] Guy HJ Prisk GK Elliott AR Deutschman RA III West JB. Inhomogeneity of pulmonary ventilation during sustained microgravity as determined by single-breath washouts. J Appl Physiol (1985) (1994) 76:1719–29. 8045852 10.1152/jappl.1994.76.4.1719

[125] Prisk GK Guy HJ Elliott AR Paiva M West JB Ventilatory inhomogeneity determined from multiple-breath washouts during sustained microgravity on Spacelab SLS-1. J Appl Physiol (1985) (1995) 78:597–607. 7759429 10.1152/jappl.1995.78.2.597

[126] Verbanck S Linnarsson D Prisk GK Paiva M Specific ventilation distribution in microgravity. J Appl Physiol (1985) (1996) 80:1458–65. 8727527 10.1152/jappl.1996.80.5.1458

[127] Prisk GK Guy HJ Elliott AR West JB. Inhomogeneity of pulmonary perfusion during sustained microgravity on SLS-1. J Appl Physiol (1985) (1994) 76:1730–8. 8045853 10.1152/jappl.1994.76.4.1730

[128] Prisk GK Fine JM Cooper TK West JB. Vital capacity, respiratory muscle strength, and pulmonary gas exchange during long-duration exposure to microgravity. J Appl Physiol (1985) (2006) 101:439–47. 10.1152/japplphysiol.01419.2005 16601306

[129] Prisk GK Elliott AR Guy HJ Kosonen JM West JB Pulmonary gas exchange and its determinants during sustained microgravity on Spacelabs SLS-1 and SLS-2. J Appl Physiol (1985) (1995) 79:1290–8. 8567575 10.1152/jappl.1995.79.4.1290

[130] Lauzon AM Elliott AR Paiva M West JB Prisk GK. Cardiogenic oscillation phase relationships during single-breath tests performed in microgravity. J Appl Physiol (1985) (1998) 84:661–8. 9475878 10.1152/jappl.1998.84.2.661

[131] Alcorn D Adamson TM Lambert TF Maloney JE Ritchie BC Robinson PM. Morphological effects of chronic tracheal ligation and drainage in the fetal lamb lung. J Anat (1977) 123:649–60. 885780 PMC1234724

[132] Moessinger AC Harding R Adamson TM Singh M Kiu GT. Role of lung fluid volume in growth and maturation of the fetal sheep lung. J Clin Invest (1990) 86:1270–7. 10.1172/JCI114834 2212011 PMC296858

[133] Newman SA Comper WD. ‘Generic’ physical mechanisms of morphogenesis and pattern formation. Development (1990) 110:1–18. 2081452 10.1242/dev.110.1.1

[134] Wolgemuth DJ Murashov AK. Models and molecular approaches to assessing the effects of the microgravity environment on vertebrate development. ASGSB Bull (1995) 8:63–72. 11538551

[135] Sanchez-Esteban J Tsai SW Sang J Qin J Torday JS Rubin LP. Effects of mechanical forces on lung-specific gene expression. Am J Med Sci (1998) 316:200–4. 10.1097/00000441-199809000-00009 9749563

[136] Torday JS Sanchez-Esteban J Rubin LP. Paracrine mediators of mechanotransduction in lung development. Am J Med Sci (1998) 316:205–8. 10.1097/00000441-199809000-00010 9749564

[137] Torday JS Rehan VK. Mechanotransduction determines the structure and function of lung and bone: a theoretical model for the pathophysiology of chronic disease. Cell Biochem Biophys (2003) 37:235–46. 10.1385/CBB:37:3:235 12625629

[138] Daifotis AG Weir EC Dreyer BE Broadus AE. Stretch-induced parathyroid hormone-related peptide gene expression in the rat uterus. J Biol Chem (1992) 267:23455–8. 1385422

[139] Curtis NE Ho PW King RG Farrugia W Moses EK Gillespie MT The expression of parathyroid hormone-related protein mRNA and immunoreactive protein in human amnion and choriodecidua is increased at term compared with preterm gestation. J Endocrinol (1997) 154:103–12. 10.1677/joe.0.1540103 9246943

[140] Steers WD Broder SR Persson K Bruns DE Ferguson JE II Bruns ME Mechanical stretch increases secretion of parathyroid hormone-related protein by cultured bladder smooth muscle cells. J Urol (1998) 160:908–12. 10.1016/S0022-5347(01)62831-3 9720586

[141] Rubin LP Torday JS Parathyroid hormone-related protein (PTHrP) biology in fetal lung development. In: Mendelson CR, editor. Endocrinology of the Lung. Totowa, NJ: Humana (2000). p. 269–97.

[142] Karaplis AC. PTHrP: novel roles in skeletal biology. Curr Pharm Des (2001) 7:655–70. 10.2174/1381612013397753 11375774

[143] Torday J Hua J Slavin R. Metabolism and fate of neutral lipids of fetal lung fibroblast origin. Biochim Biophys Acta (1995) 1254:198–206. 10.1016/0005-2760(94)00184-Z 7827125

[144] Torday JS Sun H Ling W The effect of microgravity on parathyroid hormone-related protein (PTHrP) expression by bone and lung epithelial cells. FASEB J (2000) 14:A622.

[145] Torday JS. Parathyroid hormone-related protein is a gravisensor in lung and bone cell biology. Adv Space Res (2003) 32:1569–76. 10.1016/S0273-1177(03)90397-8 15000128

[146] Clement JQ Gene expression microarrays in microgravity research: toward the identification of major space genes. In: Agbo EC, editor. Innovations in Biotechnology. Rijeka, Croatia: InTech (2012).

[147] Liu Y Wang E. Transcriptional analysis of normal human fibroblast responses to microgravity stress. Genomics Proteomics Bioinformatics (2008) 6:29–41. 10.1016/S1672-0229(08)60018-2 18558383 PMC5054092

[148] Clement JQ Lacy SM Wilson BL. Gene expression profiling of human epidermal keratinocytes in simulated microgravity and recovery cultures. Genomics Proteomics Bioinformatics (2008) 6:8–28. 10.1016/S1672-0229(08)60017-0 18558382 PMC5054098

[149] Lu WH Wang XZ Zheng Q Guan SH Xin P Sun YQ. Diversity and stability study on rice mutants induced in space environment. Genomics Proteomics Bioinformatics (2008) 6:51–60. 10.1016/S1672-0229(08)60020-0 18558385 PMC5054111

[150] Casey T Patel OV Plaut K. Transcriptomes reveal alterations in gravity impact circadian clocks and activate mechanotransduction pathways with adaptation through epigenetic change. Physiol Genomics (2015) 47:113–28. 10.1152/physiolgenomics.00117.2014 25649141

[151] Czeisler CA Chiasera AJ Duffy JF. Research on sleep, circadian rhythms and aging: applications to manned spaceflight. Exp Gerontol (1991) 26:217–32. 10.1016/0531-5565(91)90014-D 1915692

[152] Whitson PA Putcha L Chen YM Baker E. Melatonin and cortisol assessment of circadian shifts in astronauts before flight. J Pineal Res (1995) 18:141–7. 10.1111/j.1600-079X.1995.tb00152.x 7562371

[153] Fuller PM Warden CH Barry SJ Fuller CA. Effects of 2-G exposure on temperature regulation, circadian rhythms, and adiposity in UCP2/3 transgenic mice. J Appl Physiol (1985) (2000) 89:1491–8. 11007587 10.1152/jappl.2000.89.4.1491

[154] Murakami DM Fuller CA. The effect of 2G on mouse circadian rhythms. J Gravit Physiol (2000) 7(3):79–85. 12124188

[155] Robinson EL Fuller CA. Gravity and thermoregulation: metabolic changes and circadian rhythms. Pflugers Arch (2000) 441:R32–8. 10.1007/s004240000329 11200977

[156] Hilder TL Baer LA Fuller PM Fuller CA Grindeland RE Wade CE Insulin-independent pathways mediating glucose uptake in hindlimb-suspended skeletal muscle. J Appl Physiol (1985) (2005) 99:2181–8. 10.1152/japplphysiol.00743.2005 16099889

[157] Jirtle RL Skinner MK. Environmental epigenomics and disease susceptibility. Nat Rev Genet (2007) 8:253–62. 10.1038/nrg2045 17363974 PMC5940010

[158] Hayashi K Sasamura H Nakamura M Azegami T Oguchi H Sakamaki Y KLF4-dependent epigenetic remodeling modulates podocyte phenotypes and attenuates proteinuria. J Clin Invest (2014) 124:2523–37. 10.1172/JCI69557 24812666 PMC4089466

[159] Singh KP Kumari R Dumond JW. Simulated microgravity-induced epigenetic changes in human lymphocytes. J Cell Biochem (2010) 111:123–9. 10.1002/jcb.22674 20506542

[160] Chowdhury B Seetharam A Wang Z Liu Y Lossie AC Thimmapuram J A study of alterations in DNA epigenetic modifications (5mC and 5hmC) and gene expression influenced by simulated microgravity in human lymphoblastoid cells. PLoS One (2016) 11:e0147514. 10.1371/journal.pone.0147514 26820575 PMC4731572

[161] Ou X Long L Zhang Y Xue Y Liu J Lin X Spaceflight induces both transient and heritable alterations in DNA methylation and gene expression in rice (*Oryza sativa* L.). Mutat Res (2009) 662:44–53. 10.1016/j.mrfmmm.2008.12.004 19135069

[162] Alberts B The cytoskeleton. In: Alberts B Johnson A Lewis J Raff M Roberts K Walter P, editors. Molecular Biology of the Cell. New York: Garland Science (2007). p. 903–82.

[163] Wang N Butler JP Ingber DE. Mechanotransduction across the cell surface and through the cytoskeleton. Science (1993) 260:1124–7. 10.1126/science.7684161 7684161

[164] Maniotis AJ Chen CS Ingber DE. Demonstration of mechanical connections between integrins, cytoskeletal filaments, and nucleoplasm that stabilize nuclear structure. Proc Natl Acad Sci U S A (1997) 94:849–54. 10.1073/pnas.94.3.849 9023345 PMC19602

[165] Eckes B Dogic D Colucci-Guyon E Wang N Maniotis A Ingber D Impaired mechanical stability, migration and contractile capacity in vimentin-deficient fibroblasts. J Cell Sci (1998) 111(Pt 13):1897–907. 9625752 10.1242/jcs.111.13.1897

[166] Ingber DE. The architecture of life. Sci Am (1998) 278:48–57. 10.1038/scientificamerican0198-48 11536845

[167] Hubmayr RD Shore SA Fredberg JJ Planus E Panettieri RA Jr Moller W Pharmacological activation changes stiffness of cultured human airway smooth muscle cells. Am J Physiol (1996) 271:C1660–8. 8944650 10.1152/ajpcell.1996.271.5.C1660

[168] Pourati J Maniotis A Spiegel D Schaffer JL Butler JP Fredberg JJ Is cytoskeletal tension a major determinant of cell deformability in adherent endothelial cells? Am J Physiol (1998) 274:C1283–9. 9612215 10.1152/ajpcell.1998.274.5.C1283

[169] Chicurel ME Singer RH Meyer CJ Ingber DE. Integrin binding and mechanical tension induce movement of mRNA and ribosomes to focal adhesions. Nature (1998) 392:730–3. 10.1038/33719 9565036

[170] Wang N Ingber DE. Control of cytoskeletal mechanics by extracellular matrix, cell shape, and mechanical tension. Biophys J (1994) 66:2181–9. 10.1016/S0006-3495(94)81014-8 8075352 PMC1275944

[171] Wang N Ingber DE. Probing transmembrane mechanical coupling and cytomechanics using magnetic twisting cytometry. Biochem Cell Biol (1995) 73:327–35. 10.1139/o95-041 8703406

[172] Yoshida M Westlin WF Wang N Ingber DE Rosenzweig A Resnick N Leukocyte adhesion to vascular endothelium induces E-selectin linkage to the actin cytoskeleton. J Cell Biol (1996) 133:445–55. 10.1083/jcb.133.2.445 8609175 PMC2120789

[173] Tagawa H Wang N Narishige T Ingber DE Zile MR Cooper GT. Cytoskeletal mechanics in pressure-overload cardiac hypertrophy. Circ Res (1997) 80:281–9. 10.1161/01.RES.80.2.281 9012750

[174] Papaseit C Pochon N Tabony J. Microtubule self-organization is gravity-dependent. Proc Natl Acad Sci U S A (2000) 97:8364–8. 10.1073/pnas.140029597 10880562 PMC26953

[175] Tabony J Rigotti N Glade N Cortes S. Effect of weightlessness on colloidal particle transport and segregation in self-organising microtubule preparations. Biophys Chem (2007) 127:172–80. 10.1016/j.bpc.2007.01.010 17321031

[176] Lewis ML Reynolds JL Cubano LA Hatton JP Lawless BD Piepmeier EH. Spaceflight alters microtubules and increases apoptosis in human lymphocytes (Jurkat). FASEB J (1998) 12:1007–18. 9707173 10.1096/fasebj.12.11.1007

[177] Yang F Dai Z Tan Y Li Y Effects of altered gravity on the cytoskeleton of neonatal rat cardiocytes. *Micro-grav*. Sci Tech (2010) 22:45–52. 10.1007/s12217-008-9103-7

[178] Hughes-Fulford M Lewis ML. Effects of microgravity on osteoblast growth activation. Exp Cell Res (1996) 224:103–9. 10.1006/excr.1996.0116 8612673

[179] Meloni MA Galleri G Pani G Saba A Pippia P Cogoli-Greuter M. Space flight affects motility and cytoskeletal structures in human monocyte cell line J-111. Cytoskeleton (Hoboken) (2011) 68:125–37. 10.1002/cm.20499 21246756

[180] Meyers VE Zayzafoon M Gonda SR Gathings WE Mcdonald JM. Modeled microgravity disrupts collagen I/integrin signaling during osteoblastic differentiation of human mesenchymal stem cells. J Cell Biochem (2004) 93:697–707. 10.1002/jcb.20229 15660414

[181] Buravkova LB Gershovich PM Gershovich JG Grigor’ev AI. Mechanisms of gravitational sensitivity of osteogenic precursor cells. Acta Naturae (2010) 2:28–36. 22649626 PMC3347546

[182] Meyers VE Zayzafoon M Douglas JT Mcdonald JM. RhoA and cytoskeletal disruption mediate reduced osteoblastogenesis and enhanced adipogenesis of human mesenchymal stem cells in modeled microgravity. J Bone Miner Res (2005) 20:1858–66. 10.1359/JBMR.050611 16160744 PMC1351020

[183] Sciola L Cogoli-Greuter M Cogoli A Spano A Pippia P. Influence of microgravity on mitogen binding and cytoskeleton in Jurkat cells. Adv Space Res (1999) 24:801–5. 10.1016/S0273-1177(99)00078-2 11542625

[184] Hall A. Rho GTPases and the actin cytoskeleton. Science (1998) 279:509–14. 10.1126/science.279.5350.509 9438836

[185] Etienne-Manneville S Hall A. Rho GTPases in cell biology. Nature (2002) 420:629–35. 10.1038/nature01148 12478284

[186] Louis F Deroanne C Nusgens B Vico L Guignandon A. RhoGTPases as key players in mammalian cell adaptation to microgravity. Biomed Res Int (2015) 2015:747693. 10.1155/2015/747693 25649831 PMC4310447

